# Supplementary material for: Prostaglandin Metabolome Profiles in Zebrafish (Danio rerio) Exposed to Acetochlor and Butachlor
Source: Int J Mol Sci. 2023 Feb 9;24(4):3488. doi: 10.3390/ijms24043488 (PMC9963763; doi:10.3390/ijms24043488)
Supplement: Supplementary file 1 [file ijms-24-03488-s001.zip › Supplementary figures.pdf]

Figure S1: (A-B) Total ions current (TIC) overlapping map of QC samples mass spectrometry results: (A) in positive ion mode and (B) in negative ion mode.

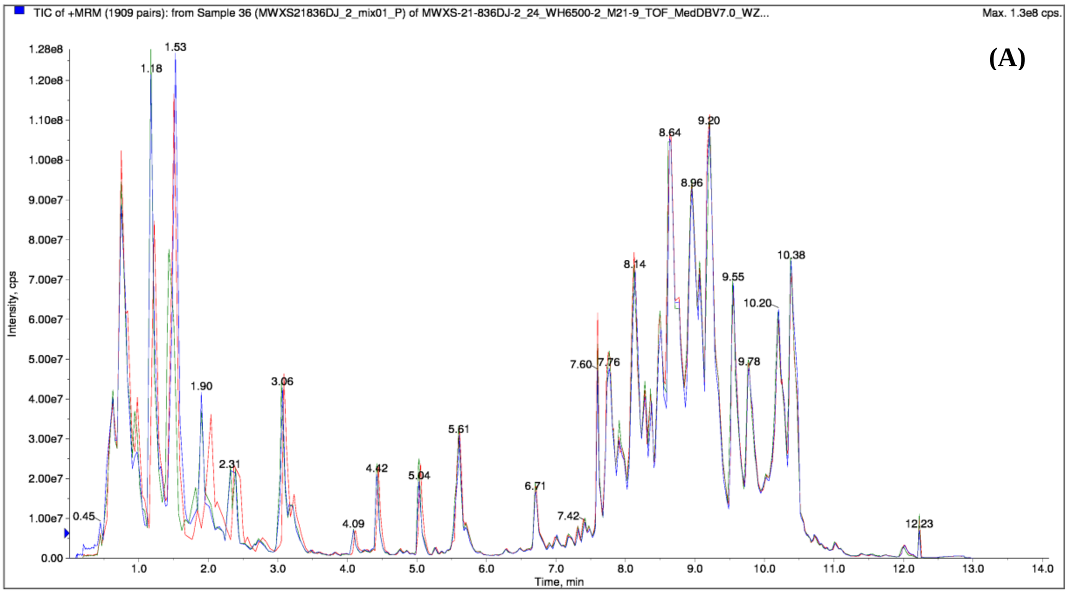

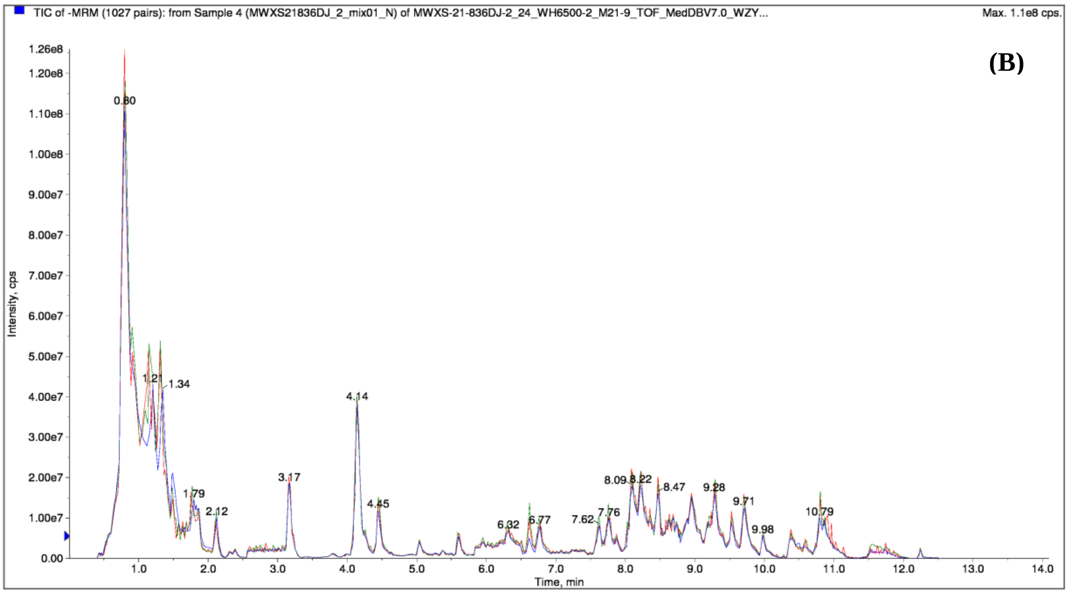

Figure S2: Multipeak map of multiple reaction monitoring (MRM) metabolite detection (ion current spectrum of multi-material extraction, XIC): (A) in positive ion mode and (B) in negative ion mode.

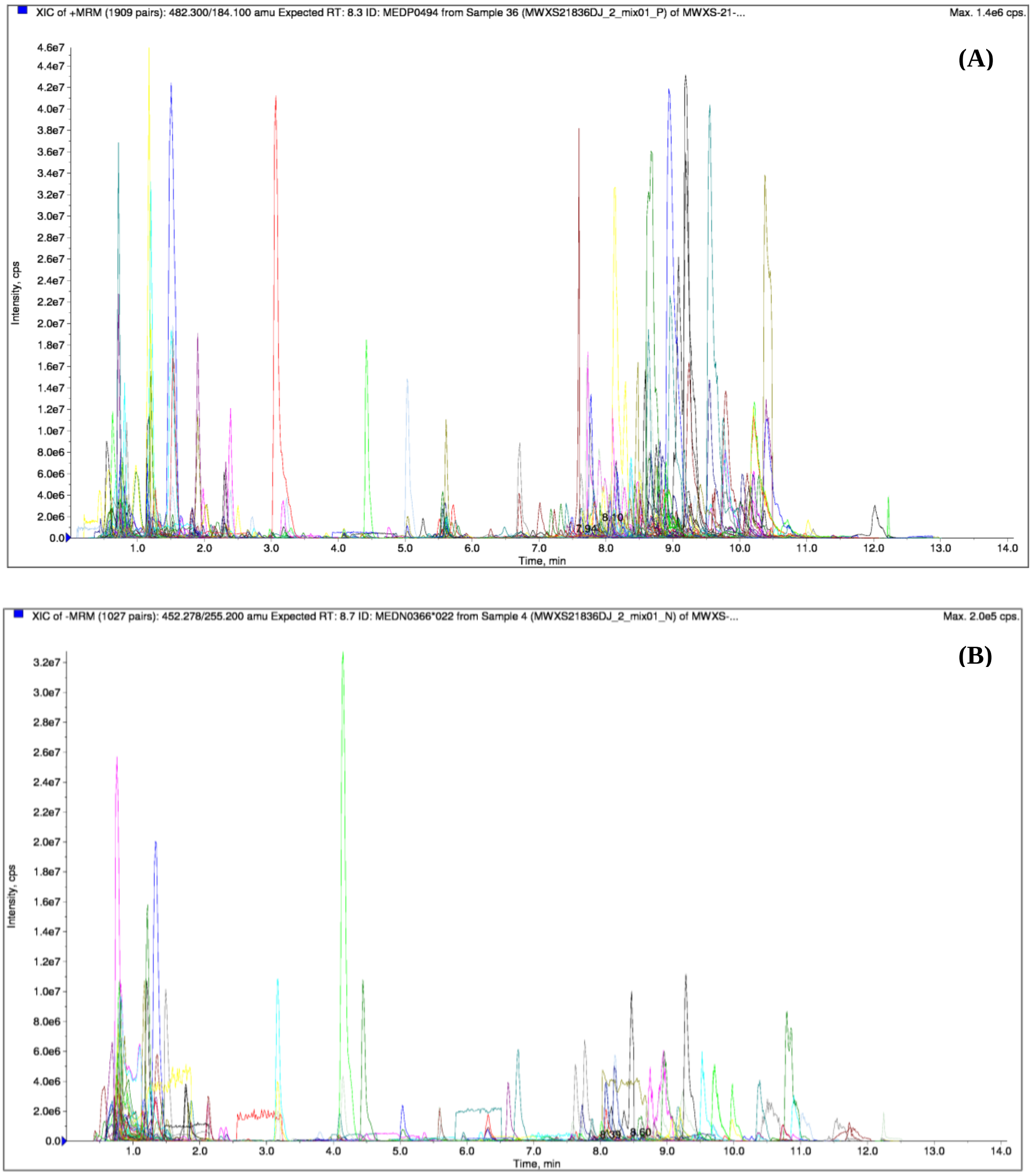

Figure S3: Orthogonal partial least squares-discriminant analysis (OPLS-DA) results showing a strong separation between each treatment group and the control group. Each point in the plot corresponds to one plasma sample. (A) ACF vs. CKF; (B) BCF vs. CKF; (C) ACM vs. CKM; (D) BCM vs. CKM.

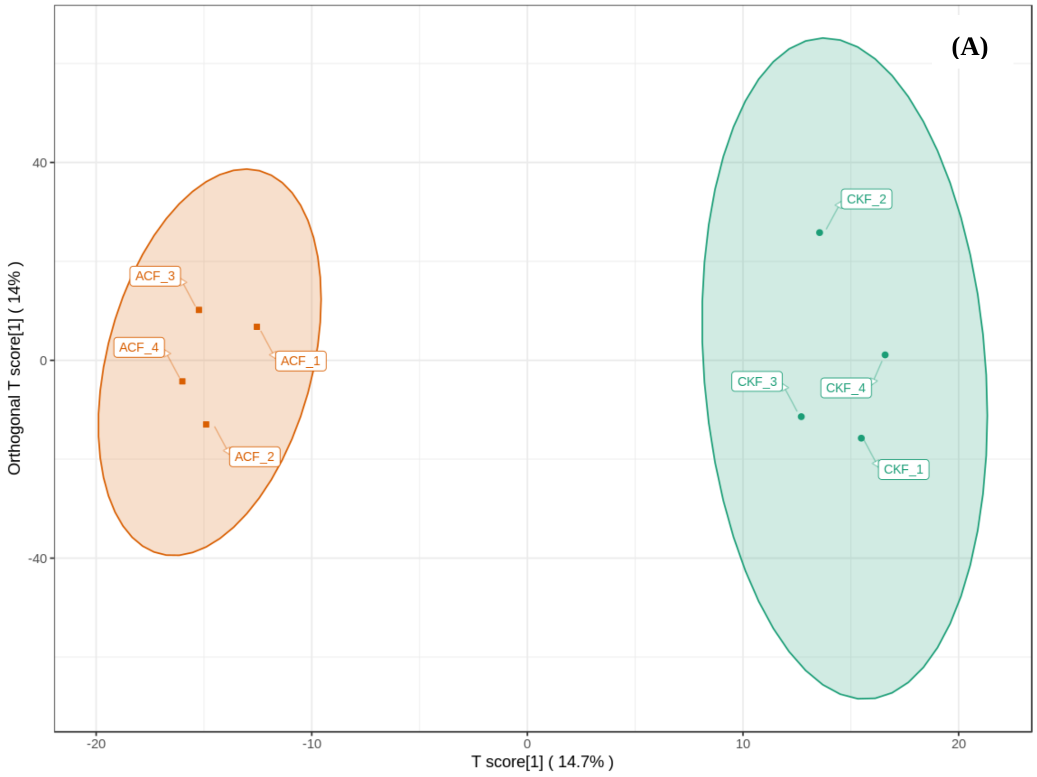

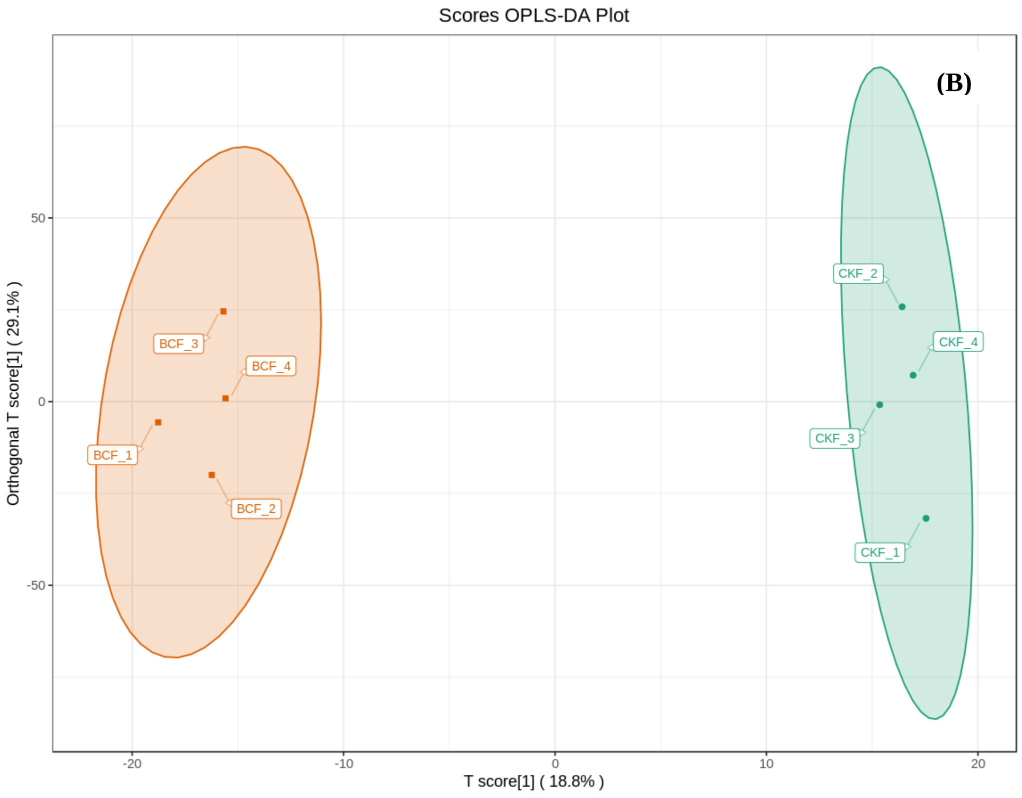

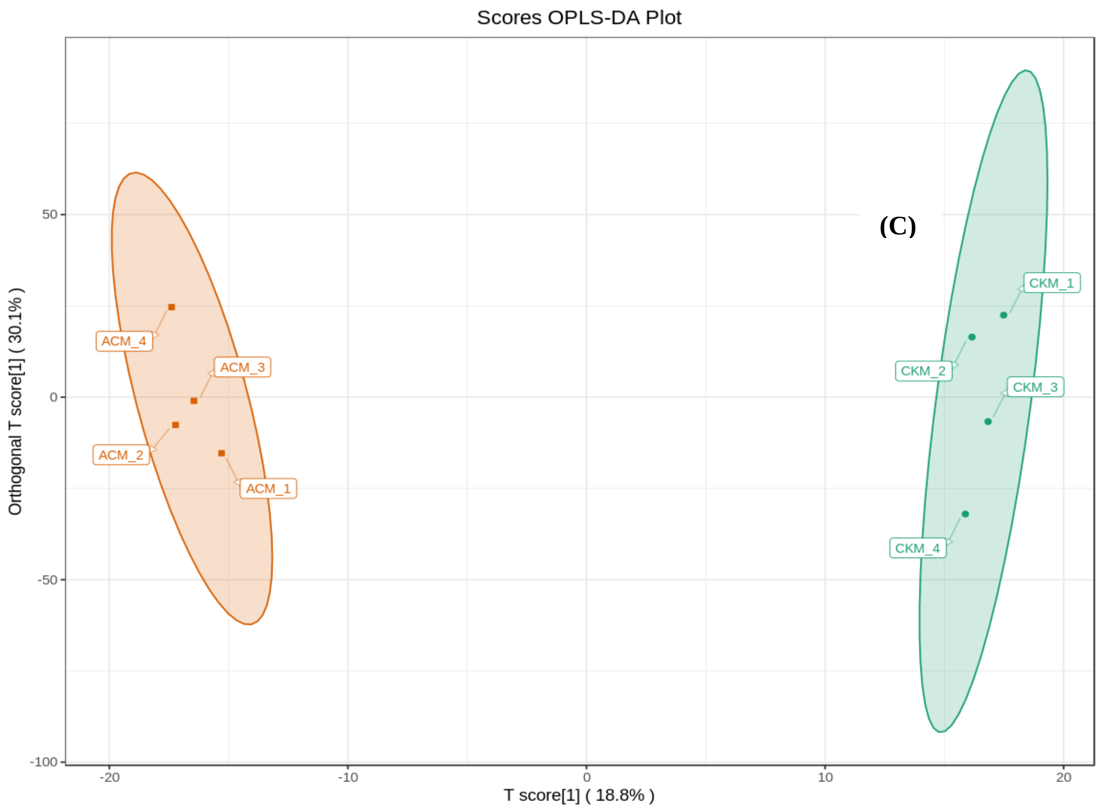
Scores OPLS-DA Plot
(C)
ACM_4
ACM_3
ACM_2
ACM_1
CKM_1
CKM_2
CKM_3
CKM_4
Orthogonal T score[1] (30.1%)
T score[1] (18.8%)
50
0
-50
-100
-20
-10
0
10
20

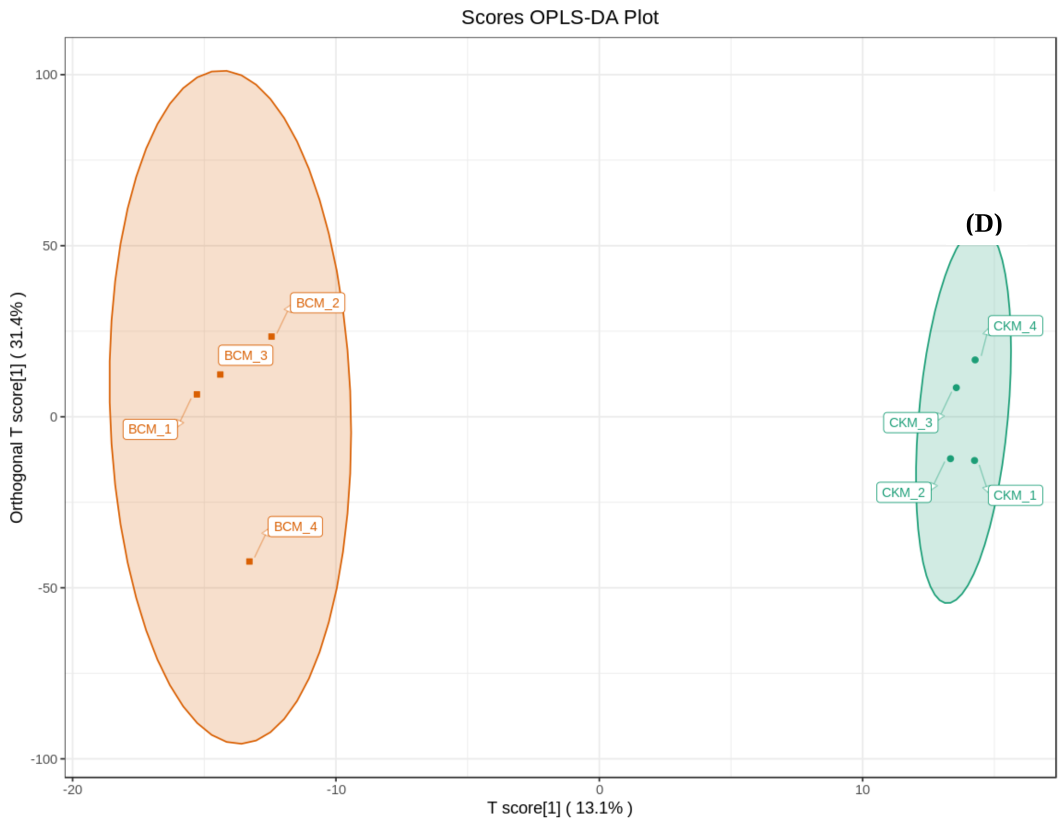
Scores OPLS-DA Plot
(D)
BCM_2
BCM_3
BCM_1
BCM_4
CKM_4
CKM_3
CKM_2
CKM_1
Orthogonal T score[1] (31.4%)
T score[1] (13.1%)
100
50
0
-50
-100
-20
-10
0
10
